# Kv4.2-Positive Domains on Dendrites in the Mouse Medial Geniculate Body Receive Ascending Excitatory and Inhibitory Inputs Preferentially From the Inferior Colliculus

**DOI:** 10.3389/fnins.2021.740378

**Published:** 2021-09-29

**Authors:** Hisataka Fujimoto, Eiji Notsu, Ryo Yamamoto, Munenori Ono, Hiroyuki Hioki, Megumu Takahashi, Tetsufumi Ito

**Affiliations:** ^1^Department of Anatomy, Kawasaki Medical School, Kurashiki, Japan; ^2^Department of Ophthalmology, Kawasaki Medical School, Kurashiki, Japan; ^3^Department of Physiology, Kanazawa Medical University, Uchinada, Japan; ^4^Department of Neuroanatomy, Juntendo University Graduate School of Medicine, Tokyo, Japan; ^5^Department of Neuroscience, Graduate School of Medicine, Kyoto University, Kyoto, Japan; ^6^Research and Education Program for Life Science, University of Fukui, Fukui, Japan; ^7^Department of Anatomy, Kanazawa Medical University, Uchinada, Japan; ^8^Department of Systems Function and Morphology, Graduate School of Innovative Life Science, University of Toyama, Toyama, Japan

**Keywords:** auditory information processing, ventral division of medial geniculate body, voltage-dependent potassium 4 proteins, reticular thalamic nucleus, inferior colliculus

## Abstract

The medial geniculate body (MGB) is the thalamic center of the auditory lemniscal pathway. The ventral division of MGB (MGV) receives excitatory and inhibitory inputs from the inferior colliculus (IC). MGV is involved in auditory attention by processing descending excitatory and inhibitory inputs from the auditory cortex (AC) and reticular thalamic nucleus (RTN), respectively. However, detailed mechanisms of the integration of different inputs in a single MGV neuron remain unclear. Kv4.2 is one of the isoforms of the Shal-related subfamily of potassium voltage-gated channels that are expressed in MGB. Since potassium channel is important for shaping synaptic current and spike waveforms, subcellular distribution of Kv4.2 is likely important for integration of various inputs. Here, we aimed to examine the detailed distribution of Kv4.2, in MGV neurons to understand its specific role in auditory attention. We found that *Kv4.2* mRNA was expressed in most MGV neurons. At the protein level, Kv4.2-immunopositive patches were sparsely distributed in both the dendrites and the soma of neurons. The postsynaptic distribution of Kv4.2 protein was confirmed using electron microscopy (EM). The frequency of contact with Kv4.2-immunopositive puncta was higher in vesicular glutamate transporter 2 (VGluT2)-positive excitatory axon terminals, which are supposed to be extending from the IC, than in VGluT1-immunopositive terminals, which are expected to be originating from the AC. VGluT2-immunopositive terminals were significantly larger than VGluT1-immunopositive terminals. Furthermore, EM showed that the terminals forming asymmetric synapses with Kv4.2-immunopositive MGV dendritic domains were significantly larger than those forming synapses with Kv4.2-negative MGV dendritic domains. In inhibitory axons either from the IC or from the RTN, the frequency of terminals that were in contact with Kv4.2-positive puncta was higher in IC than in RTN. In summary, our study demonstrated that the Kv4.2-immunopositive domains of the MGV dendrites received excitatory and inhibitory ascending auditory inputs preferentially from the IC, and not from the RTN or cortex. Our findings imply that time course of synaptic current and spike waveforms elicited by IC inputs is modified in the Kv4.2 domains.

## Introduction

The medial geniculate body (MGB) is the predominant auditory sector in the thalamus. The thalamus receives sensory information from the lower brainstem centers and modulates and sends this information to the cortex (Sherman and Guillery, 2002). Thus, the MGB relays neural information of the sound to the auditory cortex (AC) and segregates speech signals into different patterns for comprehension of the conveyed messages, interpretation of emotions associated with speech, and identification of the individual speaking (Dagnino-Subiabre et al., 2009; Bartlett, 2013; Chen et al., 2019; Kommajosyula et al., 2019; Mihai et al., 2019). In the auditory information processing, such as in language interpretation, high timing resolution is important (Dagnino-Subiabre et al., 2009), and bottom-up inputs from the inferior colliculus (IC) preserve temporal information of sound. However, if the sound is less salient, prediction from top-down inputs becomes important (Kommajosyula et al., 2019). MGB neurons must have mechanisms for the integration of the top-down and bottom-up inputs. In this aspect, the mismatch of temporal resolution along the IC–MGB–AC pathway could be a problem: the neuronal synchronization limit becomes lower in the AC than in the IC and intermediate in the MGB (Asokan et al., 2021). MGB neurons must accommodate IC inputs faster and those from AC slower.

Afferents to the MGB mainly arise from the IC, reticular thalamic nucleus (RTN), and AC (Yamada et al., 2000; Fujimoto et al., 2017; Beebe et al., 2018). Of these, the IC conveys the ascending auditory information, and the MGB receives both excitatory and inhibitory inputs from the IC (Ito et al., 2009). The MGB is subdivided into three regions: medial, ventral, and dorsal, each with distinct morphological characteristics (Hu, 2003; Anderson and Linden, 2011). The patterns of excitatory and inhibitory inputs to the MGB subdivisions also differ (Lee and Sherman, 2010). The ventral division of the MGB (MGV) shows tonotopy and is considered as the main auditory pathway (Wenstrup, 1999; Mihai et al., 2019).

The Shal-related subfamily of potassium voltage-gated channel (Kv4) forms A-type, fast-inactivation K^+^ channels that are activated or deactivated at subthreshold membrane potential values and recover with a quicker timing resolution than other Kv channels, which is important during auditory information processing (Adamson et al., 2002). Three distinct proteins are encoded by the respective genes (*Kv4.1*, *Kv4.2*, and *Kv4.3*), and the expression of *Kv4* mRNA isoforms in the rat brain has been investigated previously (Serôdio and Rudy, 1998). The mRNAs of *Kv4.2* and *Kv4.3* isoforms are abundantly expressed in the brain in what is believed to be a complementary fashion, whereas the level of *Kv4.1* mRNA expression in the brain is low. Furthermore, both *Kv4.2* and *Kv4.3* transcripts have been observed in the MGB (Serôdio and Rudy, 1998), although the resolution of the labeling was limited due to the methodology used. Kv4.2 is involved in numerous neuronal functions, and its dysfunction or mutations have been associated with disorders of the nervous system (Lin et al., 2018; Lindroos et al., 2018). Since Kv4.2 is important for shaping synaptic current and spike waveforms (Yuan et al., 2005), subcellular distribution of Kv4.2 is likely important for integration of various inputs. However, the protein expression of Kv4.2 in the MGB and its subcellular distribution have not been clarified.

Because the micromorphological details of Kv4.2 expression and excitatory and inhibitory inputs in the MGV have not yet been definitively elucidated, in the current study, we focused on the detailed distribution of Kv4.2 at the protein and mRNA levels and the involvement of Kv4.2 in synaptic organization in mouse MGV.

## Materials and Methods

### Animals

All experimental procedures were approved by the Animal Research Committees of Fukui University, Kanazawa Medical University, and the Committee of Ethics on Animal Experiments of the Kawasaki Medical School (approval #16-087). The procedures were conducted following the guidelines of the National Institutes of Health Guide for the Care and Use of Laboratory Animals (NIH Publication No. 80-23, Rev, 1996).

Eighteen male C57BL/6 mice, five adult male CBA/J mice (purchased from CLEA Japan, Inc., Tokyo, Japan), six male PV-Cre mice (Tanahira et al., 2009; a gift from Dr. Nobuaki Tamamaki, Kumamoto University, Japan), and nine adult VGAT-Cre mice of both sexes (Stock No. 016962, Jackson Laboratory, Bar Harbor, ME, United States) were used in this study. Mice were housed in standard laboratory cages with a 12-h light/dark cycle, constant temperature (24 ± 1°C), and *ad libitum* access to food and water.

### Preparation of AAV2/5 Vector for Parvalbumin Neuron Labeling

To specifically label parvalbumin (PV) neurons, we used the adeno-associated virus (AAV) vector plasmid, pAAV2-CMV-FLEX-palGFP-WPRE (Suzuki et al., 2015). The vector expresses green fluorescent protein (GFP) with a palmitoylation signal derived from the GAP-43 N-terminus (palGFP) (Moriyoshi et al., 1996; Tamamaki et al., 2000; Furuta et al., 2001; Hioki et al., 2009) in the presence of Cre recombinase. The production and purification of AAV particles were performed as previously described (Sohn et al., 2017), with several modifications. Briefly, the AAV vector and two helper plasmids, pHelper (Stratagene, La Jolla, CA, United States) and pBSIISK-R2C5, were co-transfected into HEK293T cells (RCB2202, RIKEN BRC, Japan) using polyethyleneimine. pBSIISK-R2C5 was newly prepared by inserting the following fusion sequences into the XhoI/NotI sites of pBlueScript II SK (+) (pBSIISK; Stratagene): (1) nucleotides 146–2,202 of the wild-type AAV2 genome (GenBank accession number, AF043303.1); (2) nucleotides 2,207–4,381 of AAV5 (AF085716.1); and (3) nucleotides 4,411–4,534 of AAV2. After collecting the cells, virus particles were extracted by three cycles of freezing and thawing, purified from a crude lysate of the cells by ultracentrifugation with OptiPrep (AXS-1114542; Axis-Shield, Oslo, Norway), and then concentrated by ultrafiltration with Amicon Ultra-15 Centrifugal Filter Unit with Ultracel-50 membrane (UFC905024; Merck Millipore, Darmstadt, Germany). The virus titer was adjusted to 1.0 × 10^11^ (infectious unit/mL) with Dulbecco’s phosphate-buffered saline (PBS) (14249-95; Nacalai Tesque, Kyoto, Japan), and the virus solution was stored in aliquots at −80°C until use for delivery to brain tissues.

### Injection of AAV2/5 Vector Into PV-Cre Mice

PV-Cre mice were anesthetized using a mixture of ketamine (97.6 mg/kg, i.m.) and xylazine (2.4 mg/kg, i.m.). After the animals were positioned in a stereotaxic apparatus (SR-6M; Narishige, Tokyo, Japan), a craniotomy was performed in the parietal bone, and a glass micropipette filled with AAV2/5-CMV-FLEX-palGFP-WPRE viral solution was advanced into the IC. The viral solution (0.1–1 μL) was pressure-injected using nitrogen gas and a custom-made device. After a survival period of 14 days, the mice were deeply anesthetized intraperitoneally using sodium pentobarbital (120 mg/kg) and perfused transcardially with PBS (pH 7.4; FUJIFILM Wako) followed by a mixture of 4.0% paraformaldehyde in 0.1 M phosphate buffer (PB, pH 7.4). The brains were removed from the skull after 1–2 h *in situ* at room temperature.

### Injection of AAV-FLEX-GCaMP7f Into VGAT-Cre Mice

VGAT-Cre mice were anesthetized by intraperitoneal injection of mixture of medetomidine (0.3 mg/kg), midazolam (4.0 mg/kg), and butorphanol (5.0 mg/kg) and fixed with a stereotaxic apparatus. Craniotomy was performed on the occipital and parietal bones for IC and RTN injections, respectively, and a glass pipette filled with AAV9-hSyn-FLEX-GCaMP7f (104492, Addgene, Watertown, MA, United States) was advanced into the IC or RTN. The viral solution [200 nL; the original stock was diluted in PBS 10 mM (1:5)] was slowly injected with a programmable injector (Nanoject III, Drummond Scientific, Broomall, PA, United States). After the injection, the animals received a subcutaneous injection of atipamezole (0.375 mg/kg) to facilitate recovery and were returned to their home cage. After a survival period of 14 days, the animals were deeply anesthetized by intraperitoneal injection of pentobarbital sodium (150 mg/kg) and perfused transcardially with 4% paraformaldehyde in 0.1 M PB (pH 7.4).

### Sparse Labeling of Medial Geniculate Body Neurons Using AAV-Cre, AAV-FLEX-TVA-GFP-oG, and EnvA-G-Deleted Rabies mCherry Viruses

Mice were anesthetized by intraperitoneal injection of mixture of medetomidine (0.3 mg/kg), midazolam (4.0 mg/kg), and butorphanol (5.0 mg/kg) and fixed with a stereotaxic apparatus. Craniotomy was performed on the parietal bone, and a glass pipette filled with retrograde AAV-Ef1a-Cre (Salk Institute Vector Core, La Jolla, CA, United States) was advanced into the AC. The viral solution (300 nL) was slowly injected with a programmable injector (Nanoject III). Then, a glass pipette filled with AAV8-hSyn-FLEX-TVA-P2A-eGFP-2A-oG (Salk Institute Vector Core) was advanced into the MGB, and the viral solution (500 nL) was slowly injected with a programmable injector (Nanoject III). After the injection, the animals received a subcutaneous injection of atipamezole (0.375 mg/kg) to facilitate recovery and were returned to their home cage. Fourteen days later, the animals were anesthetized again, fixed, and injected with EnvA G-deleted Rabies-mCherry (500 nL; Salk Institute Vector Core). After a survival period of 7 days, the animals were deeply anesthetized by intraperitoneal injection of pentobarbital sodium (150 mg/kg) and perfused transcardially with 4% paraformaldehyde in 0.1 M PB (pH 7.4).

### *Phaseolus vulgaris*-Leucoagglutinin Injection Into Inferior Colliculus

Three adult male mice received a unilateral iontophoretic injection of 2.5% *Phaseolus vulgaris*-leucoagglutinin (PHAL; Vector Laboratories, Burlingame, CA, United States) diluted in PBS (pH 7.2). Glass micropipettes were used to inject the PHAL iontophoretically into the IC using an intermittent current (5 mA; 10 s on/off time for 20 min). After a survival period of 14 days, the mice were deeply anesthetized intraperitoneally with sodium pentobarbital (120 mg/kg) and perfused transcardially with PBS (pH 7.4) followed by a mixture of 4.0% paraformaldehyde in 0.1 M PB (pH 7.4).

### Immunohistochemistry and Immunofluorescence

The fixed brain blocks were immersed in 30% sucrose for cryoprotection and cut into 40-μm coronal sections with a cryostat (CM1900; Leica Microsystems, Wetzlar, Germany). Every fifth section was incubated overnight with 1.0% BSA, 0.3% Triton X-100, and 0.05% NaN_3_ in PBS at 20°C. Then, the sections were incubated for 3 days at 20°C with the following primary antibodies: mouse monoclonal anti-Kv4.2 antibody (1:2,500; NeuroMab, UC Davis, United States), guinea pig polyclonal anti-vesicular glutamate transporter 2 (VGluT2) antibody (1:5,000; Frontier Institute, Ishikari, Hokkaido, Japan), guinea pig polyclonal anti-VGluT1 antibody (1:5,000; Frontier), rabbit polyclonal anti-VGluT2 antibody (1:5,000; Abcam, Cambridge, United Kingdom), rabbit polyclonal anti-glutamic acid decarboxylase (GAD) 65/67 antibody (1:5,000; Sigma-Aldrich, St Louis, MO, United States), rabbit polyclonal anti-calbindin antibody (1:2,500; Sigma-Aldrich), goat polyclonal anti-calretinin antibody (1:2,500; Abcam), chicken polyclonal anti-GFP antibody (1:2,500; Abcam), and mouse monoclonal anti-neuronal nuclei (NeuN) antibody (1:5,000; Millipore, Billerica, MA, United States). Next, sections were washed with PBS and incubated for 12 h at room temperature with a mixture of appropriate dye-conjugated secondary antibodies: biotin-conjugated goat anti-chicken antibody (1:500; Millipore, Billerica, MA, United States), FITC-conjugated donkey anti-rabbit IgG (1:500; Jackson ImmunoResearch, West Grove, PA, United States), Cy3-conjugated donkey anti-mouse IgG (1:500; Jackson ImmunoResearch), Alexa Fluor 647-conjugated donkey anti-rabbit IgG (1:500; Jackson ImmunoResearch), Alexa Fluor 647-conjugated donkey anti-goat IgG (1:500; Jackson ImmunoResearch), and Alexa Fluor 647-conjugated donkey anti-guinea pig IgG (1:500; Jackson ImmunoResearch) for 12 h at 20°C. Fluorescent Nissl counterstain was performed using NeuroTrace 435/455 (1:500; Thermo Fisher Scientific, Waltham, MA, United States). Sections treated with biotin-conjugated secondary antibody were washed with PBS and further incubated with CF405S-conjugated streptavidin (1:300; Biotium, Hayward, CA, United States) at 20°C for 12 h. Next, the sections were washed with PBS and mounted in Vectashield (Vector Laboratories). Images were acquired using a confocal laser-scanning microscope (LSM700, Carl Zeiss) with a dry objective lens (×10, NA 0.25 × 20, NA 0.8), an oil-immersion objective lens (×63, NA 1.40), and a digital microscope (BZ-X700, KEYENCE, Osaka, Japan) with a dry objective lens (×10, NA 0.25).

Quantification of the terminals, labeled by KV4.2, GAD65/67, VGluT1, and/or VGluT2 and/or GFP, was performed using the image analysis software ImageJ 1.52 (NIMH, Bethesda, MD, United States). In each mouse, three sections, including the MGV, were randomly selected, and three stacks of optical sections (60 images, *z*-interval 0.5 μm) were randomly obtained. The terminal contact is defined as within 0.5 μm location of the maximum fluorescent signal location. We randomly selected high magnification images of MGV and counted all the puncta labeled with presynaptic markers (i.e., VGluT1, VGluT2, and GFP-positive terminals with GAD65/67 immunoreactivity). We calculated the ratio between the number of presynaptic marker-positive puncta that made contacts with Kv4.2-positive structures and the total number of presynaptic marker-positive puncta. The number of images was designed to ensure sufficient statistical power. The area of VGluT1/VGluT2 fluorescently labeled puncta was measured at the maximum diameter in the stack, as the area with over 20% fluorescent intensity compared to the maximum intensity in the puncta. Different sections were used for counting the number and measuring the area of VGluT1/VGluT2 fluorescently labeled puncta.

### Fluorescence *in situ* Hybridization

Fluorescence *in situ* hybridization was performed according to a previously published protocol (Fujimoto et al., 2017). Briefly, a digoxigenin (DIG)-labeled cRNA probe was designed for the simultaneous detection of multiple mRNAs. cDNA fragments of mouse Kv4.2 (182-2080; NCBI Reference Sequence BC054462, prepared by Dr. Masahiko Watanabe, Hokkaido University) were subcloned into the pBlueScript II plasmid vector (Addgene, Cambridge, MA, United States). DIG or fluorescein-labeled cRNA probes were transcribed *in vitro* for 2 h at 37°C using linearized plasmid, DIG or fluorescein 10× RNA-labeling mix (Roche Applied Science, Mannheim, Germany), and T3 or T7 RNA polymerase (Promega, Madison, WI, United States). Next, the brain sections were subjected to acetylation, prehybridization, hybridization, post-hybridization washing, and incubation at room temperature in NaCl-Tris-EDTA buffer, 20 mM iodoacetamide in NTE buffer, and Tris-NaCl-Tween buffer as described previously (Fujimoto et al., 2017).

For immunohistochemical DIG detection, sections were blocked with DIG blocking solution [1% blocking reagent (Roche Diagnostics, Basel, Switzerland) in TNT buffer and 4% normal sheep serum] for 30 min and 0.5% TSA blocking reagent (PerkinElmer Life and Analytical Sciences, Waltham, MA, United States) in TNT buffer for 1 h. Sections were then incubated with peroxidase-conjugated anti-DIG antibody (1:500, Roche Diagnostics) for 2 h. After two TNT washes for 15 min each, fluorescence detection was performed using the Cy3-TSA plus amplification kit (PerkinElmer Life and Analytical Sciences).

### Electron Microscopy

Three C57BL/6J mice were anesthetized with a combination of sodium pentobarbital/medetomidine (50 mg/kg body weight and 0.3 mg/kg, respectively, i.p.) and perfused transcardially with a fixative solution of 4% paraformaldehyde and 1% glutaraldehyde in 0.1 M PB. The MGVs were serially cut into 50 μm coronal sections. The sections were blocked and incubated with mouse anti-Kv4.2 antibody (1:2,500; NeuroMab) in blocking solution at 4°C for 5 days. Next, the sections were rinsed with PBS and incubated with biotinylated donkey anti-mouse IgG (1:200, Jackson ImmunoResearch) in blocking solution at 20°C for 2 h and Alexa Fluor 594 FluoroNanogold-conjugated streptavidin (1:200, Nanoprobes, Yaphank, NY, United States Cat #7316, RRID:AB_2315780) in blocking solution for 2 h at 20°C. Gold immunoparticle staining was performed using a silver enhancement kit (HQ silver, Nanoprobes) at room temperature for 4 min in the dark. Next, the sections were post-fixed with 3% glutaraldehyde in 0.1 M PB for 30 min, treated with 1% osmium tetroxide in 0.1 M PB for 30 min at 4°C, and then washed with dH_2_O. The sections were incubated with 2% aqueous uranyl acetate for 30 min at 4°C and washed with dH_2_O. Finally, the sections were subjected to graded ethanol series dehydration and propylene oxide infiltration and flat-embedded in Epon-Araldite. Thin 70-nm sections were cut from these samples with an ultramicrotome (Reichert-Nissei Ultra-Cuts, Leica, Germany) and imaged with a digital transmission electron microscope (EM) (JEM-1400, JEOL, Tokyo, Japan). The area of the axon terminal profile was measured for each asymmetric synapse, with or without gold immunoparticle at the postsynaptic structure, as the cross-sectional area at the sectional level of synapse formation as previously described (Bartlett et al., 2000).

### Statistical Analysis

Data distributions were assessed for normality using the Shapiro–Wilk test. Following the assumption that all data on outcomes of interest followed a parametric distribution, a two-sample independent *t*-test was used. We judged as statistically significant when *P*-values were less than 0.05. We performed *post hoc* power analysis and confirmed that the statistical power was higher than 80%. All analyses were conducted using SPSS version 25.0 (IBM Corp., Released 2017. IBM SPSS Statistics for Windows, version 25.0. Armonk, NY: IBM Corp., United States).

## Results

### *Kv4.2* mRNA Transcript Expression in Medial Geniculate Body Neurons

**FIGURE 1:**
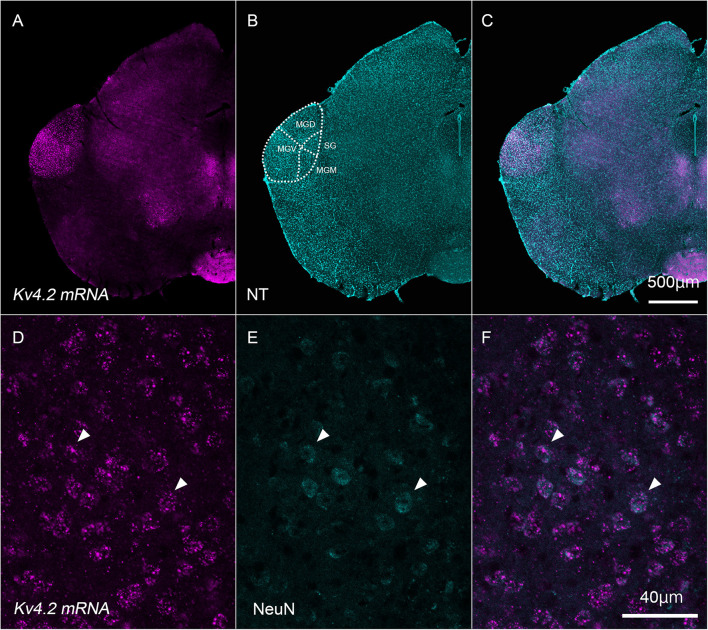
*Kv4.2* mRNA expression in neurons that have their cell bodies in the medial geniculate body. **(A–C)** Double fluorescence staining for *Kv4.2* using *in situ* hybridization (*Kv4.2* mRNA, magenta) and NeuroTrace 435/455 (NT, cyan) labeling in the adult mice brainstem coronal section, including the dorsal medial geniculate (MGD) and ventral medial geniculate (MGV). **(D–F)** *Kv4.2* subcellular localization in the MGV. High magnification of the MGB images showing double fluorescence staining of Kv4.2 using *in situ* hybridization (*Kv4.2* mRNA, magenta: **D**), immunostaining of NeuN (cyan: **E**), and merged **(F)** images. Scale bars: 500 μm **(A–C)** and 40 μm **(D–F)**. Kv4, Shal-related subfamily of potassium voltage-gated channel; MGB, medial geniculate body; MGD, dorsal MGB; MGM, medial MGB; MGV, ventral MGB; NT, NeuroTrace; NeuN, neuronal nuclei; SG, suprageniculate nucleus.

First, we examined whether *Kv4.2* mRNA was expressed in neurons whose cell bodies were within the MGB in wild-type C57BL/6 mice. To this end, we performed *in situ* hybridization and confirmed that the *Kv4.2* transcript was expressed in cells whose bodies were within the MGB (Figure 1). Indeed, *Kv4.2* mRNA was strongly expressed in the MGB but not in the neighboring brain areas (Figures 1A–C). Within the MGB, MGV showed stronger expression of Kv4.2 than dorsal and medial division (MGD and MGM) and suprageniculate nucleus (SG) did (Figure 1A). In the MGV, almost all of the neurons (99.7%; 759/761 cells measured in three mice), which were labeled with a neuronal marker, NeuN, co-expressed *Kv4.2* mRNA (Figures 1D–F), confirming the expression of Kv4.2 in MGV neurons.

### Kv4.2 Protein Is Present in the Medial Geniculate Body of the Thalamus

**FIGURE 2:**
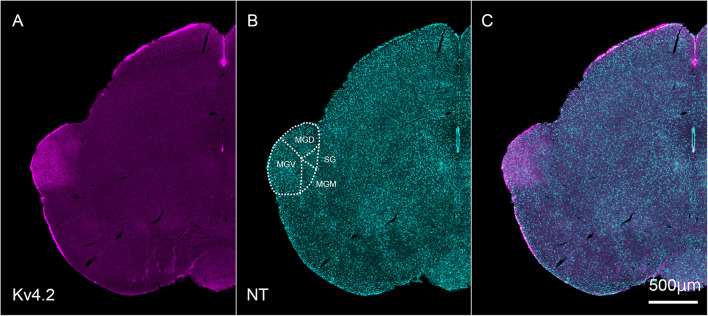
Kv4.2 protein expression in the MGB examined by immunohistochemistry. **(A–C)** Double fluorescence for voltage-dependent potassium channel 4.2 (Kv4.2, magenta: **A**), NeuroTrace 435/455 (NT, cyan: **B**), and merged **(C)** labeling in the adult mouse brainstem coronal section, including MGB. Scale bar: 500 μm. MGB, medial geniculate body; MGD, dorsal MGB; MGM, medial MGB; MGV, ventral MGB; NT, NeuroTrace; SG, suprageniculate nucleus.

Next, we tested whether Kv4.2 protein was present in the MGB. Using immunofluorescence, we confirmed that Kv4.2 was expressed in the MGB of the thalamus and showed a diffuse pattern in wild-type C57BL/6 mice (Figure 2). Based on the cytoarchitecture, which was revealed by NeuroTrace fluorescent Nissl staining, Kv4.2 expression was strictly localized to the MGB, and almost no expression was observed in other neighboring areas (Figure 2).

We examined the subcellular distribution of Kv4.2 protein in the CBA/J mice MGB neurons using sparse labeling of MGB neurons using AAV-Cre, AAV-FLEX-TVA-GFP-oG, and EnvA G-deleted Rabies mCherry viruses (see section “Materials and Methods”). We found that Kv4.2 immunoreactivity was distributed as sparse patches on both the proximal and distal dendrites and the soma of MGV neurons (Supplementary Figure 1).

**FIGURE 3:**
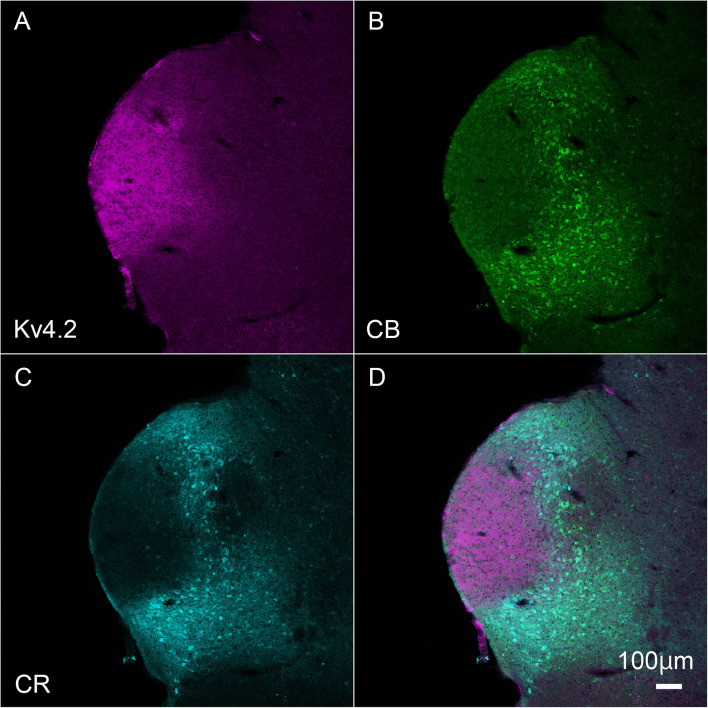
Kv4.2 protein expression is mainly observed in the ventral MGB examined by immunohistochemistry. **(A–D)** Triple fluorescence labeling for voltage-dependent potassium channel 4.2 (Kv4.2, magenta: **A**), calbindin (CB, Green: **B**), calretinin (CR, cyan: **C**), and merged **(D)** images in the adult mouse brainstem coronal section including MGV. Scale bar: 100 μm.

Inside the MGB, Kv4.2 immunoreactivity appeared stronger in MGV than in MGD, MGM, and SG. It has been shown that MGV neurons lacked calretinin (CR) and calbindin (CB) proteins (Cruikshank et al., 2001; Lu et al., 2009), and we found that Kv4.2 protein was expressed mainly in the region in which expression of CR and CB was lacking (Figure 3).

### Kv4.2-Rich Domains Form Contacts Preferentially With Vesicular Glutamate Transporter 2 but Not With VGluT1 in Medial Geniculate Body Neurons

**FIGURE 4:**
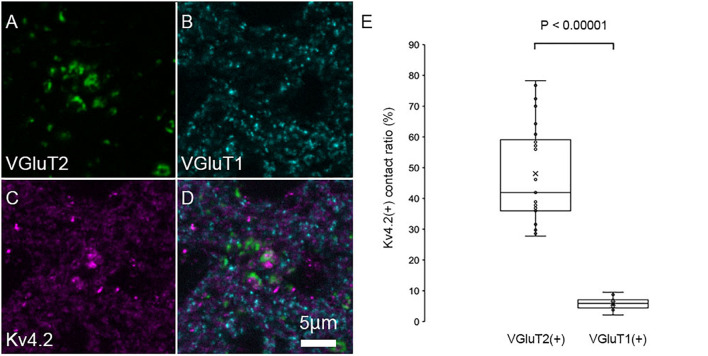
Kv4.2 protein domains are found in preferential contact with VGluT2 and not with VGluT1. **(A–D)** Triple immunofluorescent labeling for vesicular glutamate transporter 2 (VGluT2, green: **A**), vesicular glutamate transporter 1 (VGluT1, cyan: **B**), voltage-dependent potassium channel 4.2 (Kv4.2, magenta: **C**), and merged **(D)** as shown in images in the adult mouse MGV. Scale bar: 5 μm. **(E)** The ratio of (VGluT1 or VGluT2 positive) axon terminals that are contact with Kv4.2-positive puncta in all (VGluT1 or VGluT2 positive, respectively) axon terminals is significantly higher in the VGluT2-positive excitatory axon terminals than in the VGluT1-positive terminals (*P* < 0.00001, *t*-test). The horizontal lines in the box and whisker plots represent the median values, and the bottom and top of the boxes represent the lower and upper quartiles, respectively. The “x” represents the mean, and the bars represent the minimum and maximum values within 1.5 times the lower and upper quartiles.

The patchy distribution of Kv4.2 protein suggests the presence of Kv4.2-rich microdomains at the surface of MGV neurons. To investigate the relationship between synapses on MGV neurons and Kv4.2-rich microdomains, we next examined the subcellular localization of the Kv4.2 protein in relation to excitatory synapses in wild-type C57BL/6 mice. It has been shown that (1) MGB neurons do not have axon collaterals in the MGB (Bartlett and Smith, 1999; Smith et al., 2006), (2) IC excitatory neurons express VGluT2 but not VGluT1 (Ito et al., 2011), (3) neocortical excitatory neurons express VGluT1 (Fremeau et al., 2001), and (4) neurons in MGD and MGV coexpress mRNA for VGluT1 and VGluT2 (Ito et al., 2011; Hackett et al., 2016). Accordingly, we used VGluT1 and VGluT2 antibodies to label cortical and IC excitatory terminals, respectively. Upon performing triple immunofluorescent staining for VGluT1, VGluT2, and Kv4.2, we found that Kv4.2-positive domains were in preferential contact with VGluT2-positive terminals in the MGB neurons (Figures 4A–D). Colocalization of VGluT1 and VGluT2 was not observed in axon terminals, confirming that the terminals were not originated from neurons in MGV or MGD. Figure 4E shows that the ratio of the axon terminals that formed contact with Kv4.2-positive puncta was significantly higher in VGluT2-positive excitatory axon terminals (48.1 ± 15.8%, *N* = 689 puncta in 27 sections of nine mice) than in VGluT1-positive terminals (5.9 ± 1.9%, *N* = 1,629 puncta in 18 sections of nine mice) (*P* < 0.00001, *t*-test).

The area of VGluT1/VGluT2 fluorescently labeled puncta in the MGB was also examined. Since a previous study (Bartlett et al., 2000) reported that the axon terminals from the IC were larger than those from the AC in the MGB, we hypothesized that VGluT2-positive terminals are larger than VGluT1-positive terminals. Indeed, we confirmed that the area was significantly (*P* < 0.00001, *t*-test) larger in VGluT2-positive terminals (0.89 ± 0.71 μm^2^, *N* = 258 puncta in nine sections of nine mice) than in VGluT1-positive terminals (0.24 ± 0.15 μm^2^, *N* = 770 puncta in nine sections of nine mice).

**FIGURE 5:**
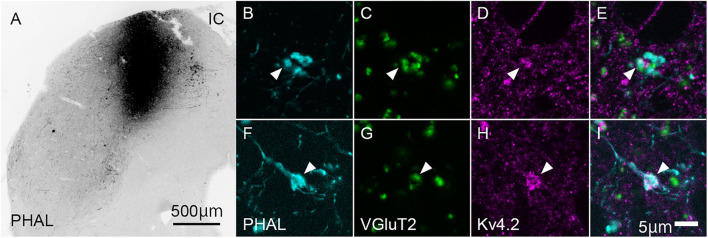
Kv4.2-positive processes from MGB neurons receive excitatory (VGluT2) inputs from the IC. **(A)** An example of the coronal section of IC showing the center of injection of *Phaseolus vulgaris*-leucoagglutinin (PHAL). **(B–I)** Triple immunofluorescence for PHAL (cyan; **B,E**), VGluT2 (green; **C,G**), and Kv4.2 (magenta; **D,H**), in the mice MGV. **(E,I)** Images are acquired from different sections. Scale bars: 500 μm **(A)**, 5 μm **(B–I)**. IC, inferior colliculus; VGluT2, vesicular glutamate transporter 2.

To confirm whether the origin of VGluT2-positive axon terminals in MGB is IC, we injected the anterograde tracer PHAL into the IC and examined its colocalization of VGluT2 in terminals originating from the IC. Indeed, the PHAL-positive axon terminals in the MGB expressed VGluT2 and made contact with Kv4.2-positive puncta (Figure 5). The ratio of colocalization of VGluT2 among PHAL-positive, IC-originated terminals was 85.8 ± 4.7%. The remaining 14% of PHAL-positive terminals were positive for GAD65/67, and PHAL-positive terminals without immunoreactivity for VGluT2 or GAD65/67 were almost absent. Among the terminals co-expressing VGluT2 and PHAL, 48.9 ± 13.2% (*N* = 554 puncta in 12 sections of three mice) of them were opposed with Kv4.2-positive domain. The percentage was similar and not significantly different from that of VGluT2-positive terminals apposed with Kv4.2-positive domain to all VGluT2-positive terminals in MGV (48.1 ± 15.8%, *P* = 0.87, *t*-test), further confirming that IC excitatory neurons are the main source of VGluT2-positive terminals in MGV and make contact on Kv4.2-positive domain.

**FIGURE 6:**
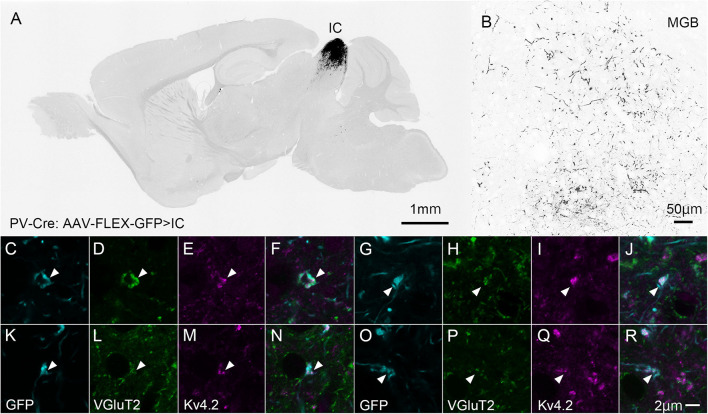
Kv4.2-positive processes from MGV neurons receive excitatory (VGluT2) inputs from the IC (GFP). **(A)** An example of a GFP-immunostained sagittal section containing IC and showing the center of injection. **(B)** GFP-positive axonal plexuses in the MGB. **(C–R)** Triple immunofluorescence for GFP (cyan; **C,G,K,O**), VGluT2 (green; **D,H,L,P**), and Kv4.2 (magenta; **E,I,M,Q**) in the MGVof PV-Cre mice. **(F,J,N,R)** Images are acquired from different sections. Scale bar: 1 mm **(A)**, 50 μm **(B)**, and 2 μm **(C–R)**. GFP, green fluorescent protein; IC, inferior colliculus; Kv4, Shal-related subfamily of potassium voltage-gated channel; MGV, ventral medial geniculate; VGluT2, vesicular glutamate transporter 2.

Ventral division of MGB is characterized by strong immunoreactivity for PV (Cruikshank et al., 2001), although it does not express PV mRNA (Allen Brain Atlas). We then labeled the source of the PV fibers. To this end, we injected the AAV2/5-FLEX-GFP vector into the IC of PV-Cre mice to label axon terminals from the IC with GFP (Figures 6A,B). Since PV is expressed in both GABAergic and glutamatergic neurons in the IC (Fujimoto et al., 2017), the terminals may colocalize VGluT2. Using this method, we examined triple immunohistochemistry for GFP, Kv4.2, and VGluT2 to determine whether Kv4.2 was expressed on the receiving side of VGluT2 excitatory inputs that arise from the IC. We found that Kv4.2-immunopositive puncta were located in close proximity to terminals that colocalized with GFP and VGluT2 (Figures 6C–R).

### Formation of Synapses Between Kv4.2-Positive Postsynaptic Medial Geniculate Body Regions and Excitatory Terminals

**FIGURE 7:**
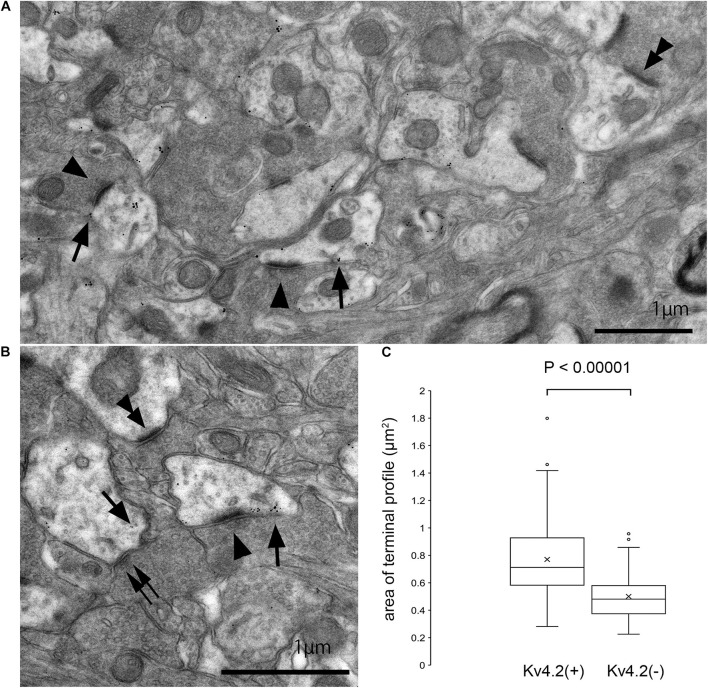
Formation of synapses between Kv4.2-positive and negative postsynaptic MGB microregions and VGluT2 excitatory terminals. **(A,B)** Examples of asymmetric excitatory synapses in the MGV using pre-embedding immunoelectron microscopy. Arrow: Kv4.2 (gold colloid), arrowhead: asymmetrical synapse that is in the contact with Kv4.2-positive postsynaptic structure, double arrowhead: asymmetrical synapse that is in the contact with Kv4.2-negative postsynaptic structure, and double arrow: symmetrical synapse that is in the contact with Kv4.2-positive postsynaptic structure. **(C)** The area of axon terminals of those asymmetric synapses that are in contact with gold particles positive or negative postsynaptic structure. Terminals forming asymmetric synapse with Kv4.2-positive MGV dendrites are significantly larger than those forming synapses with Kv4.2-negative MGV dendrites (*P* < 0.00001, *t*-test). Scale bar: 1 μm **(A,B)**. The horizontal lines in the box and whisker plots represent the median values, and the bottom and top of the boxes represent the lower and upper quartiles, respectively. The “x” represents the mean and the bars represent the minimum and maximum values within 1.5 times the lower and upper quartiles. Kv4, Shal-related subfamily of potassium voltage-gated channel; MGV, ventral medial geniculate.

The above results strongly suggest that Kv4.2-positive microdomains are postsynaptic to excitatory terminals that arise from the IC. However, since these results were obtained using light microscopy, it was difficult to judge whether the Kv4.2 puncta were pre- or postsynaptic. Therefore, we performed immunoelectron microscopy for Kv4.2 on the MGV in wild-type C57BL/6 mice, and found that the immunogold was located on the cell membranes of dendritic processes, which were postsynaptic with relatively large axon terminals filled with small round vesicles (arrowheads in Figures 7A,B) *via* asymmetric synapses. Immunogold labeling was not observed on the cell membrane of axonal terminals. These results indicate the formation of synapses between Kv4.2-expressing dendritic processes of MGV neurons and excitatory terminals (Figures 7A,B).

Next, we measured the area of axon terminals that made asymmetric synapses, and compared the areas between terminals presynaptic to dendrites with gold particles and those without. On EM, terminals making asymmetric synapse with Kv4.2-positive MGV dendrites were significantly larger (mean 0.77 ± 0.25 μm^2^, cross-sectional area, *N* = 291 synapse in nine sections of three mice) than those with Kv4.2-negative MGV dendrites (0.50 ± 0.17 μm^2^, *N* = 162 synapse in nine sections of three mice) (*P* < 0.00001, *t*-test) (Figure 7C).

### Formation of Synapses Between Kv4.2-Positive Postsynaptic MGB Regions and Inhibitory Terminals From Inferior Colliculus but Not Reticular Thalamic Nucleus

Finally, we tested the origin of inhibitory presynaptic inputs to Kv4.2-expressing dendrites. Inhibitory afferents to the MGB mainly arise from the RTN and IC (Bartlett et al., 2000). To examine the origin, we specifically labeled inhibitory axons that originated from the IC or RTN by injecting the AAV-FLEX-GCaMP7f vector into the IC or RTN of VGAT-Cre mice.

**FIGURE 8:**
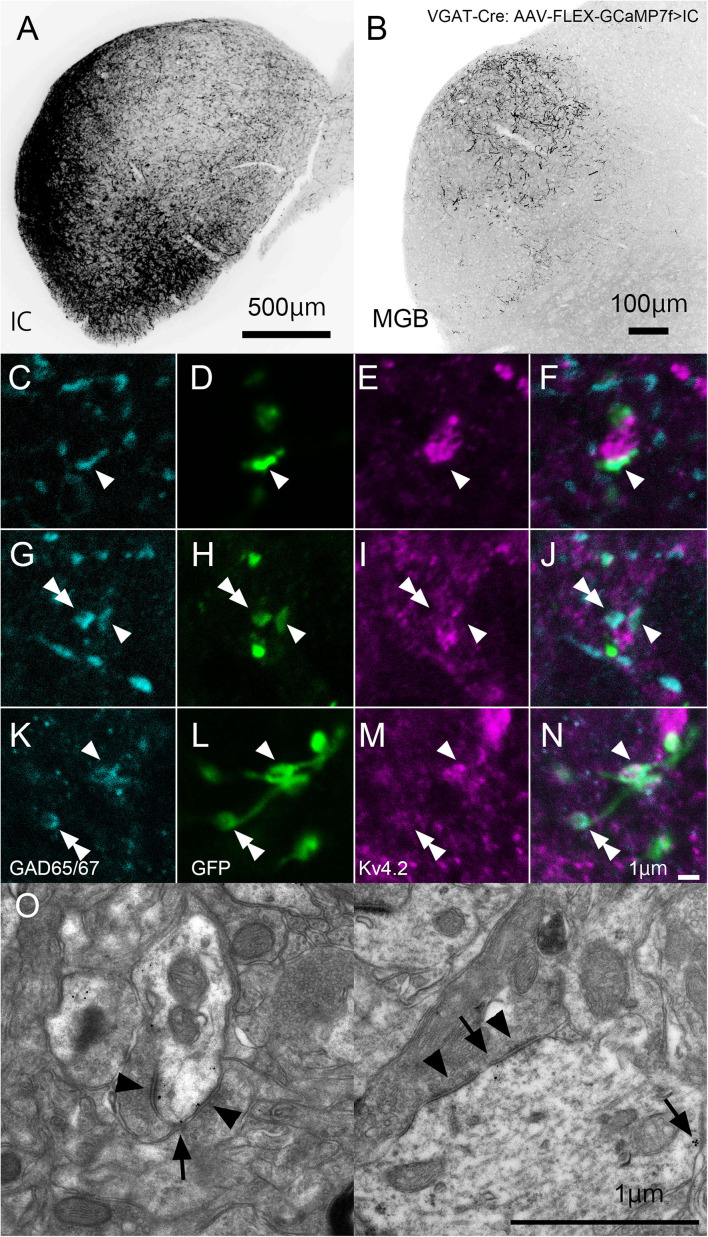
Formation of synapses between Kv4.2-positive postsynaptic MGV microregions and inhibitory terminals from IC. **(A)** An example of a GFP-immunostained coronal section of IC, showing the injection center. **(B)** GFP-positive axonal plexuses in the MGB. **(C–N)** Triple fluorescence for glutamic acid decarboxylase 65/67 (GAD65/67, cyan; **C,G,K**), GFP (green; **D,H,L**), and Kv4.2 (magenta; **E,I,M**) in the MGV of VGAT-Cre mice. Arrowhead: GAD65/67 positive terminal that is in the contact with Kv4.2-positive structure and double arrowhead: GAD65/67 positive terminal that does not make a contact with Kv4.2 positive structure. **(E,J,N)** Images are acquired from different sections. **(O)** Examples of symmetric inhibitory synapses in the MGV using pre-embedding immunoelectron microscopy. Arrow: Kv4.2 (gold colloid) and arrowhead: symmetrical synapse that is in contact with Kv4.2-positive postsynaptic structure. Scale bar: 500 μm **(A)**, 100 μm **(B)**, and 1 μm **(C–O)**. GFP, green fluorescent protein; IC, inferior colliculus; Kv4, Shal-related subfamily of potassium voltage-gated channel; MGB, medial geniculate body; MGV, ventral medial geniculate; VGAT, vesicular gamma-amino butyric acid transporter.

In IC-injected mice, the injection sites were confirmed by the strong GFP signal in the IC (Figure 8A), and dense GFP-positive axon plexuses were found in the MGV (Figure 8B). We performed triple immunohistochemistry for GFP, Kv4.2, and GAD65/67 in the MGV to determine whether Kv4.2 was expressed on the receiving side of inhibitory inputs that originated from the IC. We found that Kv4.2-positive puncta came into close proximity with GAD65/67-positive inhibitory terminals from IC neurons, which were labeled with GFP (Figures 8C–N). Furthermore, immunoelectron microscopy of Kv4.2 revealed that terminals containing pleomorphic vesicles formed symmetrical synapses on MGV dendritic processes, the cell membrane of which was labeled with Kv4.2-positive gold particles, confirming the formation of synapses between Kv4.2-expressing processes of MGV neurons and inhibitory terminals (Figure 8O).

**FIGURE 9:**
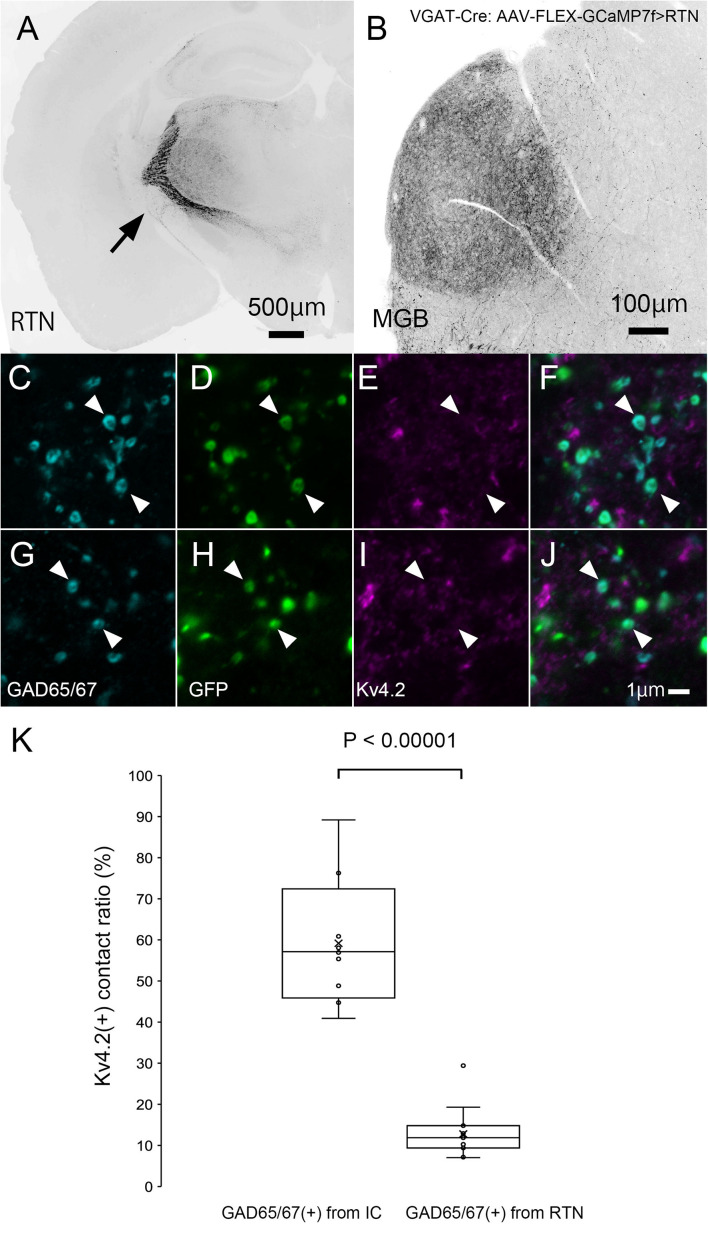
Inhibitory synapse is formed preferentially between Kv4.2-positive MGV microregions and IC axon terminals but not RTN axon terminals. **(A)** An example of a GFP-immunostained coronal section of RTN showing dense labeling of RTN neurons. **(B)** GFP-positive axonal plexuses in the MGB. **(C–J)** Triple fluorescence for GAD65/67 (cyan; **C,G**), GFP (green; **D,H**), and Kv4.2 (magenta; **E,I**) in the MGV of VGAT-Cre mice. Arrowhead: GAD65/67-positive terminal from RTN that does not make a contact with Kv4.2-positive structure. **(E,J)** Images are acquired from different sections. **(K)** The ratio of GAD65/67- and GFP-double-positive axon terminals that are in contact with Kv4.2-positive puncta in all GAD65/67- and GFP-double-positive axon terminals is higher from the IC than from RTN regions (*P* < 0.00001, *t*-test). The horizontal lines in the box and whisker plots represent the median values, and the bottom and top of the boxes represent the lower and upper quartiles, respectively. The “x” represents the mean, and the bars represent the minimum and maximum values within 1.5 times the lower and upper quartiles. Scale bar: 500 μm **(A)**, 100 μm **(B)**, and 1 μm **(C–J)**. GAD, glutamic acid decarboxylase; GFP, green fluorescent protein; Kv4, Shal-related subfamily of potassium voltage-gated channel; MGB, medial geniculate body; MGV, ventral medial geniculate; RTN, reticular thalamic nucleus; VGAT, vesicular gamma-amino butyric acid transporter.

In RTN-injected mice, the injection sites were confirmed by strong GFP labeling (Figure 9A), and dense GFP-positive plexuses were observed in the MGV (Figure 9B). In contrast with IC-injected brains, axon terminals from RTN, where GFP colocalized with GAD65/67, did not frequently come in close proximity with Kv4.2-positive puncta (Figures 9C–J). Figure 9K shows that the ratio of GAD65/67- and GFP-double-positive terminals that made contact with Kv4.2-positive puncta in all the GAD65/67- and GFP-double-positive terminals was significantly higher from the IC (59.2 ± 14.7%, *N* = 461 terminals in 12 sections of four mice) than those from RTN (12.8 ± 5.7%, *N* = 583 terminals in 15 sections of five mice) (*P* < 0.00001, *t*-test).

**FIGURE 10:**
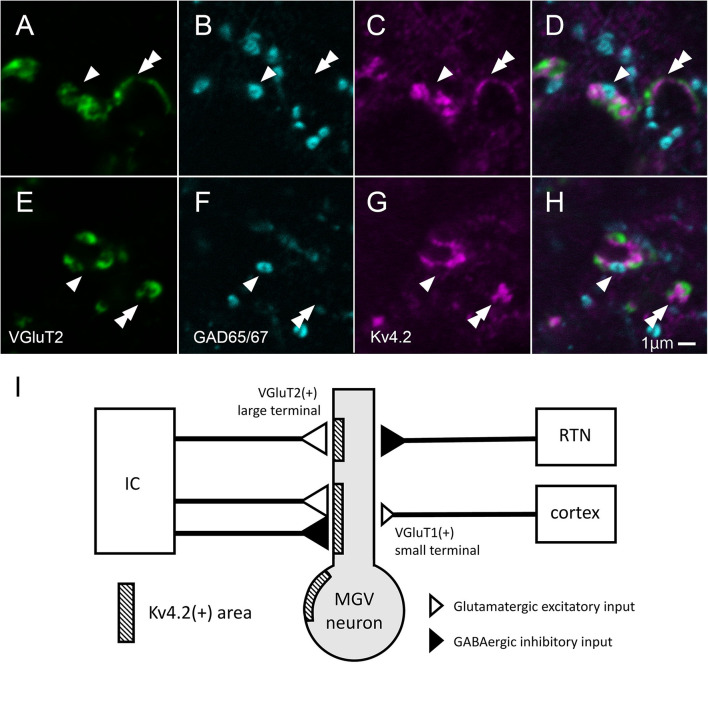
Some of Kv4.2-positive domains receive contact with both VGluT2 excitatory and GABAergic inhibitory axon terminals. **(A–H)** Triple fluorescence labeling for VGluT2 (green; **A,E**), GAD65/67 (cyan; **B,F**), and Kv4.2 (magenta; **C,G**) in the mice MGV. Arrowhead: Kv4.2-positive structure that is closely opposite to both GAD65/67-positive terminal and VGluT2-positive terminal. Double arrowhead: Kv4.2-positive structures that are closely opposite to VGluT2-positive terminals but not to GAD65/67-positive terminals. **(I)** A summary schematic diagram representing the relationship between Kv4.2-positive structures and glutamatergic and GABAergic inputs from the IC, RTN, and cortex. Scale bar: 1 μm **(A–H)**. GABA, gamma-aminobutyric acid; GAD, glutamic acid decarboxylase; IC, inferior colliculus; Kv4, Shal-related subfamily of potassium voltage-gated channel; MGV, ventral medial geniculate; RTN, reticular thalamic nucleus; VGluT2, vesicular glutamate transporter 2.

In MGV, a few Kv4.2-positive domains did not colocalize with either the VGluT2 excitatory or the GABAergic inhibitory axon terminals (Figures 10A–H). The ratio of Kv4.2-positive puncta that made contact with both the GAD65/67 positive terminal and VGluT2 positive terminals in all the Kv4.2-positive puncta was 9.6 ± 3.5% (*N* = 431 terminals in 18 sections of nine mice) in confocal microscopic observation.

## Discussion

This study established the micromorphological details of the expression pattern of Kv4.2 protein in mouse MGB in relation to synapses from extrinsic sources, namely, AC, IC, and RTN. We also discovered preferential synapse formation between Kv4.2 positive postsynaptic dendrites in MGV and both excitatory and inhibitory terminals originating from the IC, but not inhibitory terminals from the RTN. As most MGV neurons express *Kv4.2* mRNA, single MGV neurons are highly likely to possess both Kv4.2-positive domains, receiving inputs from IC, and Kv4.2-negative domains, receiving inputs from the AC and RTN (Figure 10I).

Our findings suggest that Kv4.2 plays a unique role in the auditory system. In the auditory nerve, A-type voltage-gated potassium channels are thought to adjust to electrophysiologically hasten cell responses up to 1,000 Hz of action potential, which is likely to reflect adaptation in auditory circuits that require quick reactions (Altoè et al., 2018). In our study, Kv4.2-positive MGV cell dendrites were frequently observed to be closely opposed to both VGluT2-positive (IC-derived) and IC-derived GAD-positive terminals.

Furthermore, our study revealed that these synapses had a unique neural circuit structure, in which they received both inhibitory and excitatory inputs from the same lower nucleus, the IC. An exploration of the fine structure of MGB synapses in rodents by Bartlett et al. (2000) reported the relationship between the size and origins, i.e., AC, RTN, and IC, of presynaptic terminals in the MGB. We found that VGluT2-positive, presumable IC-derived excitatory terminals were larger than VGluT1-positive, presumable cortical excitatory terminals, which was consistent with previous findings. In addition, we demonstrated that Kv4.2 was a marker of postsynaptic microdomains that preferentially received both VGluT2 excitatory and GABAergic inhibitory inputs from the IC in MGV. The inhibitory axon terminals from the RTN were forming synapses on Kv4.2-positive microdomains less frequently. The axon terminals positive for VGluT1 (and negative for VGluT2) in MGV which made contact on Kv4.2-positive domain less frequently, were possibly originated from AC.

Considering that MGB neurons do not have axon collaterals in the MGB (Bartlett and Smith, 1999; Smith et al., 2006), neurons in MGD and MGV co-express mRNA for VGluT1 and VGluT2 (Ito et al., 2011; Hackett et al., 2016), and complemental expression of VGluT1 and VGluT2 in terminals in MGV, it is less likely that VGluT1- or VGluT2-positive terminals were from MGB.

In our study, Kv4.2 was localized to the postsynaptic region in MGV neurons. Kv4.2 is likely to reduce neural excitation, when the neurons are highly depolarized (Kimm and Bean, 2014). The activation of Kv4.2 may facilitate repolarization of the membrane potential after action potential generation, resulting in a narrower spike width. At lower membrane voltages, convergence of inhibitory and excitatory inputs from the IC may cause shunting inhibition and result in a narrow postsynaptic potential. Therefore, both Kv4.2 and ascending inputs may sharpen temporal tuning. Descending inputs from AC and RTN are less likely to show the sharpening of temporal tunings as the interaction of excitatory postsynaptic potential and inhibitory postsynaptic potential because Kv4.2 is less associated with these inputs. It has been shown that the interaction of top-down and bottom-up inputs was important for predicting the temporal structures of sound in MGB neurons (Kommajosyula et al., 2019). The authors used salient (100% amplitude modulation) and less salient (25% amplitude modulation) sound sequences that were predictable or random order. They showed that, in response to less-salient stimuli, MGB neurons showed an increased preference for predictable sequence and a decreased envelope locking to amplitude modulation, suggesting that temporal features of ascending inputs are different from those of descending inputs. MGV neurons may compute information from the Kv4.2 microdomains, which integrate ascending inputs and convey temporal structure, and other dendritic domains that integrate descending inputs and carry prediction. Because the expression of Kv4.2 is weak in non-lemniscal regions of MGB (i.e., MGD, MGM, and SG), such dendritic integration is likely to be the key feature of the lemniscal pathway. Physiological studies are required for further interpretation of the function of Kv4.2 in MGB.

Overall, our study characterized the micromorphological expression of Kv4.2 in the MGB, including its excitatory and inhibitory inputs, as well as the existence of synapses involving Kv4.2-expressing neurons. Our findings may contribute to a better understanding of the micromorphological organization of the MGV and its specific role in the attention and prediction of sound.

## Data Availability Statement

The data that support the findings of this study are available from the corresponding author, HF and TI upon reasonable request.

## Ethics Statement

The animal study was reviewed and approved by the Animal Research Committees of Fukui University, the Animal Research Committees of Kanazawa Medical University, and the Committee of Ethics on Animal Experiments of the Kawasaki Medical School.

## Author Contributions

All authors had full access to all study data and were responsible for the data integrity and accuracy of the data analysis. HF: study concept and design, statistical analysis, and study supervision. HF, EN, RY, MO, and TI: data acquisition. HF and TI: analysis and interpretation of the data and drafting of the manuscript. HF, TI, and HH: funding. RY, MO, HH, and MT: administrative, technical, and material support.

## Conflict of Interest

The authors declare that the research was conducted in the absence of any commercial or financial relationships that could be construed as a potential conflict of interest.

## Publisher’s Note

All claims expressed in this article are solely those of the authors and do not necessarily represent those of their affiliated organizations, or those of the publisher, the editors and the reviewers. Any product that may be evaluated in this article, or claim that may be made by its manufacturer, is not guaranteed or endorsed by the publisher.

## Abbreviations

AAV: adeno-associated virus
DIG: digoxigenin
EPSP: excitatory postsynaptic potential
GABA: gamma-aminobutyric acid
GAD: glutamic acid decarboxylase
GFP: green fluorescent protein
IC: inferior colliculus
IPSP: inhibitory postsynaptic potential
Kv4: Shal-related subfamily of potassium voltage-gated channel
MGB: medial geniculate body
MGD: dorsal medial geniculate
MGM: medial division of the medial geniculate body
MGV: ventral medial geniculate
NeuN: neuronal nuclei
PBS: phosphate-buffered saline
PHAL: *Phaseolus vulgaris*-leucoagglutinin
PV: parvalbumin
RTN: reticular thalamic nucleus
SG: suprageniculate nucleus
VGAT: vesicular gamma-amino butyric acid transporter
VGluT1: vesicular glutamate transporter 1
VGluT2: vesicular glutamate transporter 2.
