# Supplementary material for: Kv4.2-Positive Domains on Dendrites in the Mouse Medial Geniculate Body Receive Ascending Excitatory and Inhibitory Inputs Preferentially From the Inferior Colliculus
Source: Front Neurosci. 2021 Sep 29;15:740378. doi: 10.3389/fnins.2021.740378 (PMC8511456; doi:10.3389/fnins.2021.740378)
Supplement: Supplementary file 1 [file Image_1.pdf]

Supplementary Material

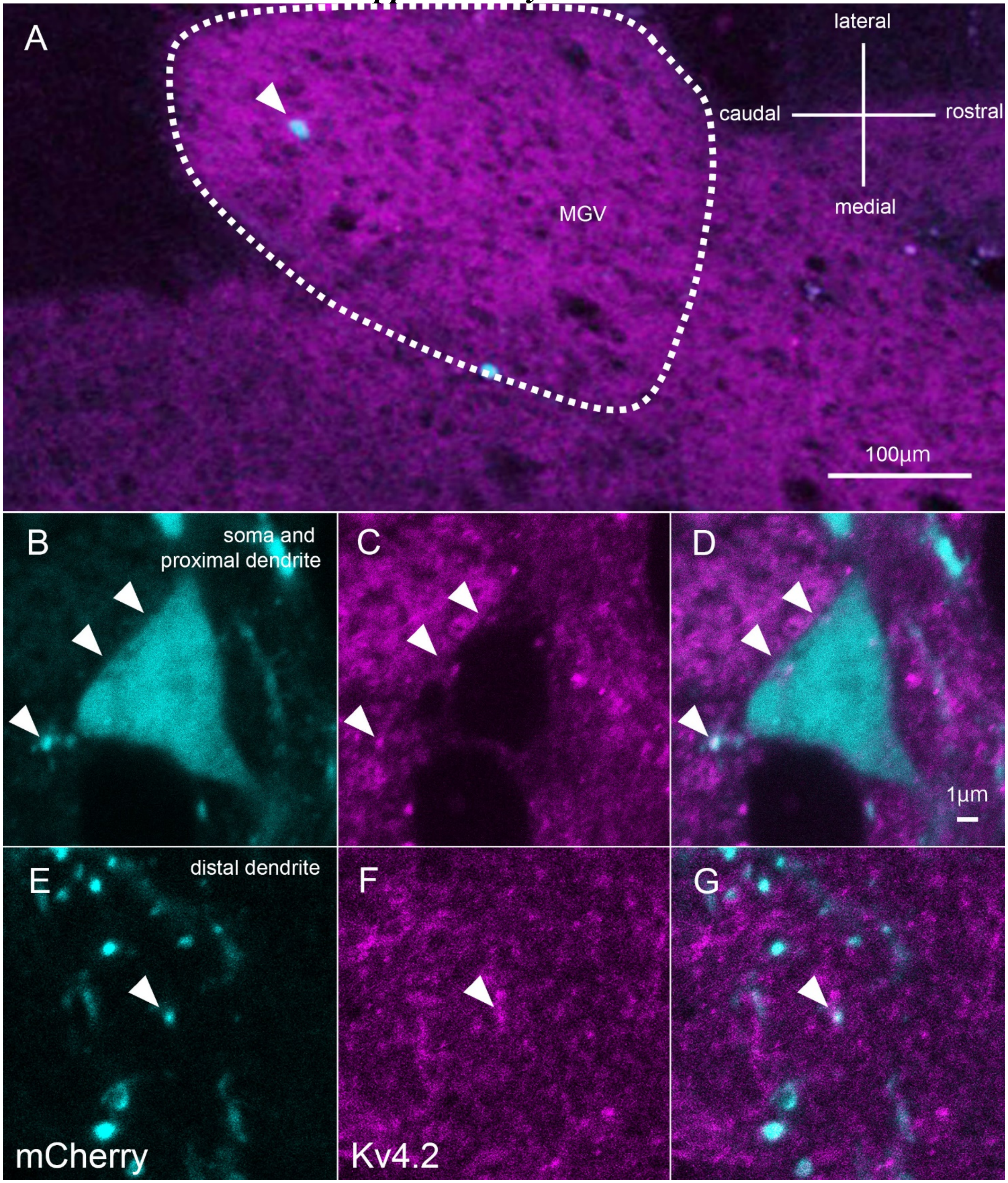

Supplementary Figure 1. Kv4.2-positive patches were sparsely distributed in both the proximal, distal dendrites and the soma of MGV neurons. A, Double fluorescence for mCherry (cyan) and Kv4.2 (magenta) in the MGV horizontal section. Using sparse labeling method with combination of rabies and helper viruses, mCherry-positive neurons (cyan) were sparsely labeled and some were inside the MGV (arrowhead), which was identified with strong Kv4.2 immunolabeling. **B-G,** Double fluorescence for mCherry (cyan; **B, G**) and Kv4.2 (magenta; **C, F**) in the MGV neuron **D** and **G** show overlay images of **B** + **C** and **E** + **F**, respectively. Scale bar: 1 μm. AAV, Adeno-associated virus; MGV, ventral medial geniculate.

.
